# Comparative Evaluation of Mineral Content and Surface Topography of Teeth After Bleaching Using Energy-Dispersive X-ray Spectroscopy (EDS) and Scanning Electron Microscopy (SEM): An In Vitro Study

**DOI:** 10.7759/cureus.117510

**Published:** 2026-10-05

**Authors:** Harshita Garg, Munish Singla, Harleen Kaur, Saloni Gupta, Deepa Dubey, Rupinder Bansal, Joshna Gupta, Mehak Gupta

**Affiliations:** 1 Conservative Dentistry and Endodontics, Adesh Institute of Dental Sciences and Research, Bathinda, IND; 2 Oral Medicine and Radiology, Adesh Institute of Dental Sciences and Research, Bathinda, IND; 3 Oral and Maxillofacial Surgery, Adesh Institute of Dental Sciences and Research, Bathinda, IND

**Keywords:** carbamide peroxide, light-activated pap, non-peroxide bleaching agents, phthalimidoperoxycaproic acid, tooth whitening oxidation

## Abstract

Objectives: This in vitro study evaluated the impact of various bleaching systems on enamel surface topography and mineral composition (calcium and phosphate) using scanning electron microscopy (SEM) and energy-dispersive X-ray spectroscopy (EDS).

Materials and methods: Forty-eight human enamel specimens (N=48) were divided into five groups: Group 1a (positive control; untreated), Group 1b (negative control; 37% phosphoric acid etching), Group 2 (37.5% hydrogen peroxide, Pola Office+, SDI, Bayswater, Victoria, Australia), Group 3 (22% carbamide peroxide, Pola Night, SDI, Bayswater, Victoria, Australia), and Group 4 (phthalimidoperoxycaproic acid (PAP), Toothsi Spark, Nanchang, Jiangxi, China). Following group-specific clinical regimens, specimens were stored in artificial saliva and evaluated using SEM-EDS. Data were analyzed via one-way analysis of variance (ANOVA) and Bonferroni tests (alpha=0.05).

Results: Calcium levels differed significantly among the five groups (one-way ANOVA; F(4,43)=27.88; p<0.001). Group 1a showed the highest mean calcium level, whereas Group 1b showed the lowest. Among the bleaching groups, Groups 2 and 4 showed significantly higher calcium levels than Group 3 on Bonferroni-adjusted post hoc comparison. Phosphorus levels did not differ significantly among the groups (one-way ANOVA; F(4,43)=0.52; p=0.720).

Conclusion: Extended application timelines render 22% carbamide peroxide more detrimental to enamel mineral content than brief, high-concentration hydrogen peroxide or light-activated PAP.

## Introduction

Modern lifestyle habits, particularly the high consumption of caffeinated beverages among younger demographics, have significantly increased the incidence of extrinsic tooth discoloration. Because a healthy smile is intrinsically linked to psychological and social well-being [1], the global demand for conservative aesthetic dentistry has expanded exponentially [2]. Dental discolorations are generally classified as extrinsic or intrinsic. Extrinsic staining stems from topical exposure to agents like coffee, tea, tobacco, and chlorhexidine rinses. Conversely, intrinsic discoloration arises from systemic factors, including fluorosis, genetic anomalies, trauma, and aging [3,4].

To meet rising aesthetic expectations without compromising healthy tissue, conservative treatments are preferred over invasive restorations like veneers or crowns [5]. Consequently, tooth bleaching remains the most widely utilized, minimally invasive cosmetic procedure [2]. Conventional bleaching relies on hydrogen peroxide or carbamide peroxide formulations. Mechanistically, these agents release highly reactive free radicals, such as hydroxyl radicals, which break down the conjugated double bonds of organic chromophores within the dental structure, shifting their light absorption spectrum to produce a whitening effect [6,7]. Carbamide peroxide acts as an indirect source; for instance, a 22% carbamide peroxide gel dissociates into urea and roughly 7.7% active hydrogen peroxide [8].

However, the powerful oxidation process driving these agents can be detrimental to the dental substrate [9]. Prolonged exposure to peroxide radicals can alter the histological and morphological integrity of enamel, inducing surface porosity, microhardness loss, and mineral depletion [10,11,7]. To mitigate these adverse effects, peroxide-free alternatives have been introduced. Phthalimidoperoxycaproic acid (PAP) is a non-radical oxygen donor that bleaches chromogens through an alternative oxidation pathway without generating aggressive free radicals [12].

While various formulations promise effective whitening, their relative impact on enamel safety remains a subject of ongoing debate. Bleaching-induced demineralization and structural degradation, specifically regarding changes in calcium/phosphate (Ca/P) ratios, vary considerably across different active ingredients [13,14]. Therefore, the aim of this in vitro study was to compare the effects of three different bleaching systems, that is, 37.5% hydrogen peroxide, 22% carbamide peroxide, and PAP, on human enamel surface morphology and calcium and phosphorus content, using scanning electron microscopy (SEM) and energy-dispersive X-ray spectroscopy (EDS), respectively.

## Materials and methods

This study was conducted at Adesh Institute of Dental Sciences and Research in Bathinda, India, after obtaining ethical approval from the Ethics Committee for Biomedical and Health Research (EC_BHR) of Adesh University, Bathinda, before its commencement (approval number: AU/EC_BHR/2K24/596; approval date: 17/05/2024).

Twenty-four sound, permanent human anterior teeth extracted due to poor periodontal prognosis were selected. Carious, fractured, restored, root canal-treated, severely fluorosed, or attrited teeth were excluded. Roots were removed at the cementoenamel junction using a low-speed diamond disc. Crowns were sectioned longitudinally into halves to obtain 48 enamel specimens (N=48).

Grouping and bleaching protocols

The specimens were randomly allocated to control and experimental groups. The control group comprised two subgroups of six specimens each, while the three experimental groups contained 12 specimens each. Group 1a (positive control; n=6) received no surface treatment. Group 1b (negative control; n=6) was etched with 37% phosphoric acid (Septodont, Saint-Maur-des-Fossés, France) for 30 seconds. Group 2 (n=12) was treated with 37.5% hydrogen peroxide (Pola Office+, SDI, Bayswater, Victoria, Australia) for three consecutive eight-minute applications, followed by rinsing and storage in 20 mL of artificial saliva. Group 3 (n=12) was treated with 22% carbamide peroxide (Pola Night, SDI, Bayswater, Victoria, Australia) for 45 minutes daily for seven consecutive days and stored in artificial saliva between treatment cycles. Group 4 (n=12) was treated with PAP (Toothsi Spark 2nd Gen, Nanchang, Jiangxi, China) for 30 minutes daily for 10 days. The first 10 minutes of each session included blue LED light activation (BlancOne Ultra+, IDS S.p.A., Savona, Italy).

SEM

Surface topography and elemental mineral composition (calcium and phosphate weight percentages) were evaluated using SEM (JEOL JSM-IT710HR SEM, JEOL Ltd., Tokyo, Japan). After vacuum desiccation and gold sputter-coating, the specimens were examined at 1,000× magnification to assess surface changes.

EDS

Mineral quantification was done using Arsenazo III reagent (Sigma-Aldrich, St. Louis, Missouri, United States); the test solution was incubated at 37°C, and absorbance was measured at 630 nm using a Shimadzu UV-1800 spectrophotometer (Shimadzu Corporation, Kyoto, Japan).

Statistical analysis

Data were analyzed using IBM SPSS Statistics for Windows, Version 21.0 (IBM Corp., Armonk, New York, United States), and expressed as mean±standard deviation. Normality was confirmed via the Shapiro-Wilk test. Intergroup differences were assessed using one-way analysis of variance (ANOVA), followed by the Bonferroni post hoc test for pairwise comparisons. Statistical significance was set at p<0.05.

## Results

The mean calcium levels varied among the five study groups. Group 1A (positive control) showed the highest mean calcium level (40.73±2.40 wt%), whereas Group 1B (negative control) showed the lowest (16.28±3.98 wt%). Among the bleaching groups, Group 2 showed the highest mean calcium level (30.32±5.05 wt%), followed by Group 4 (27.75±4.99 wt%) and Group 3 (21.20±4.76 wt%). One-way ANOVA showed a statistically significant difference in mean calcium levels among the five groups (F(4,43)=27.88; p<0.001) (Figure 1).

**Figure 1:**
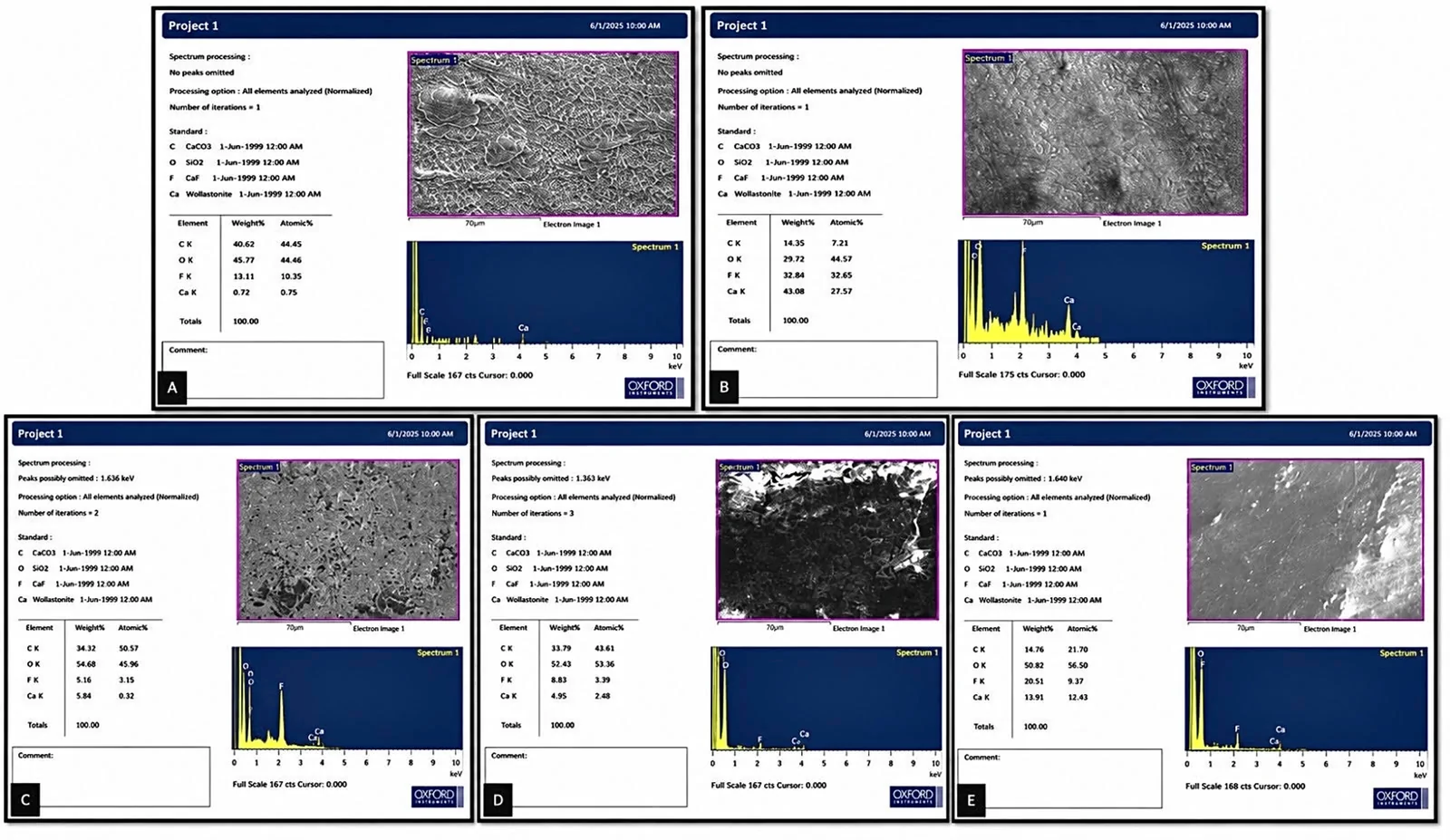
SEM: EDS report of (A) Group 1a, (B) Group 1b, (C) Group 2, (D) Group 3, and (E) Group 4 SEM: scanning electron microscopy; EDS: energy-dispersive X-ray spectroscopy

Bonferroni-adjusted post hoc pairwise comparisons showed that Group 1A had significantly higher calcium levels than Groups 1B, 2, 3, and 4 (all p<0.001). Group 1B had significantly lower calcium levels than Groups 2 and 4 (both p<0.001), whereas the difference between Groups 1B and 3 was not statistically significant (p=0.380). Group 2 showed significantly higher calcium levels than Group 3 (p<0.001), while the difference between Groups 2 and 4 was not statistically significant (p=1.000). Group 4 also showed significantly higher calcium levels than Group 3 (p=0.011). The complete pairwise comparisons are presented in Table 1.

**Table 1:** Pairwise intergroup comparisons of calcium levels Bonferroni post hoc statistical test was applied.

(I) group	(J) group	Mean difference (I-J)	Std. error	P-value	95% CI	
					Lower bound	Upper bound
Gr 1A	Gr 1B	24.45167	2.65783	<0.001	16.5867	32.3166
	Gr 2	10.40417	2.30175	<0.001	3.5929	17.2154
	Gr 3	19.52417	2.30175	<0.001	12.7129	26.3354
	Gr 4	12.97583	2.30175	<0.001	6.1646	19.7871
Gr 1B	Gr 2	-14.04750	2.30175	<0.001	-20.8587	-7.2363
	Gr 3	-4.92750	2.30175	0.380	-11.7387	1.8837
	Gr 4	-11.47583	2.30175	<0.001	-18.2871	-4.6646
Gr 2	Gr 3	9.12000	1.87937	<0.001	3.5587	14.6813
	Gr 4	2.57167	1.87937	1.000	-2.9897	8.1330
Gr 3	Gr 4	-6.54833	1.87937	0.011	-12.1097	-0.9870

The mean phosphorus concentrations showed relatively little variation among the study groups. Group 1a demonstrated the highest mean phosphorus concentration (23.07±6.09 wt%), whereas Group 1b showed the lowest mean value (18.92±4.76 wt%). The mean phosphorus concentrations were 21.69±6.12 wt% in Group 2, 19.87±6.17 wt% in Group 3, and 20.85±5.64 wt% in Group 4 (Figure 1).

One-way ANOVA showed no statistically significant difference in mean phosphorus concentrations among the five groups (F(4,43)=0.52; p=0.720). Similarly, Bonferroni-adjusted pairwise comparisons showed no statistically significant differences between any of the groups (all p>0.05). The complete pairwise comparisons are presented in Table 2.

**Table 2:** Pairwise intergroup comparisons of phosphorus levels Bonferroni post hoc statistical analysis was applied.

(I) group	(J) group	Mean difference (I-J)	Std. error	P-value	95% CI	
					Lower bound	Upper bound
Gr 1A	Gr 1B	4.14500	3.38552	0.737	-5.4932	13.7832
	Gr 2	1.37250	2.93195	0.990	-6.9744	9.7194
	Gr 3	3.19083	2.93195	0.812	-5.1561	11.5377
	Gr 4	2.21250	2.93195	0.942	-6.1344	10.5594
Gr 1B	Gr 2	-2.77250	2.93195	0.877	-11.1194	5.5744
	Gr 3	-0.95417	2.93195	0.997	-9.3011	7.3927
	Gr 4	-1.93250	2.93195	0.964	-10.2794	6.4144
Gr 2	Gr 3	1.81833	2.39392	0.941	-4.9969	8.6336
	Gr 4	0.84000	2.39392	0.997	-5.9752	7.6552
Gr 3	Gr 4	-0.97833	2.39392	0.994	-7.7936	5.8369

## Discussion

Enamel is the most highly mineralized and hardest tissue in the human body, composed primarily of hydroxyapatite crystals packed into a dense, low-porosity structure. Despite its structural resilience, the modern rise in aesthetic demand, heavily amplified by mass media and evolving beauty standards, has made conservative tooth whitening a highly sought-after dental procedure [2]. Because anterior teeth are highly visible during smiling, they are the primary targets for cosmetic enhancements. However, the prolonged contact required during whitening exposes the outer enamel surface to powerful oxidative processes, raising significant clinical concerns regarding enamel wear, microhardness loss, and structural degradation [15,16].

To evaluate these structural risks, this study utilized SEM and EDS. SEM is well-established as a high-resolution tool for characterizing microscopic alterations in hard tissue topography [17]. However, EDS weight percentages represent the relative elemental composition of the analyzed area; they do not directly measure the absolute amount of mineral removed, phosphate ions, or enamel mechanical properties. The calcium and phosphorus outcomes should therefore be interpreted separately, and no conclusion about the Ca/P ratio can be drawn because that ratio was not analyzed. To establish a clear standard for demineralization, a negative control group (Group 1b) was etched with 37% phosphoric acid for 30 seconds. This process selectively dissolves hydroxyapatite crystals, creating a distinctive microporous surface against which the experimental bleaching protocols could be benchmarked [14].

An unexpected finding in this study was that Group 3 (22% carbamide peroxide; Pola Night) demonstrated the greatest loss of calcium and phosphate ions, along with the most pronounced topographic alterations under SEM, even when compared to the higher concentration hydrogen peroxide group (Group 2). These findings describe between-group differences after treatment. Because the reported design did not include specimen-specific pretreatment EDS measurements, it cannot establish the magnitude of calcium reduction within the individual specimens. The untreated control provides a reference but does not eliminate potential baseline differences between teeth. This outcome can be explained by the kinetics of the bleaching protocols. As documented by Karimi et al., enamel decalcification is a function of both active agent concentration and total exposure time. While Pola Night utilizes a lower equivalent hydrogen peroxide concentration (~7.7%), it requires a prolonged daily application of 45 minutes over seven days [18]. This extended contact time generates cumulative oxidative stress, leading to deeper chemical diffusion and a more pronounced breakdown of the enamel matrix compared to shorter regimens [19,20].

Conversely, Group 2 (37.5% hydrogen peroxide; Pola Office+) demonstrated the highest mineral retention and least surface disruption among the bleached groups. This favorable outcome is likely due to its minimal application time lapse (three eight-minute cycles in a single session) and its neutral pH (pH=7), which mitigates the severe, low-pH acid erosions frequently observed with older peroxide formulations [21].

Interestingly, the intergroup comparison between Group 2 (hydrogen peroxide) and Group 4 (PAP) yielded non-significant differences in mineral changes. Mechanistically, these two agents operate via distinct pathways: hydrogen peroxide relies on the aggressive generation of reactive oxygen free radicals, whereas PAP utilizes a non-radical epoxidation mechanism to break down organic chromophores [22]. Bypassing radical formation theoretically grants PAP a safer clinical profile [23]. However, in this study, the PAP (Toothsi Spark) required a 30-minute daily exposure for 10 days, paired with blue LED light activation. Light activation accelerates oxidation kinetics, increasing the velocity of chromophore breakdown [24]. Consequently, the sustained oxidative activity of PAP over a longer clinical timeline, amplified by light energy, delivered a cumulative oxidative impact that mirrored the structural effects of a brief, high-concentration peroxide application.

The untreated control had the highest reported calcium content (40.73±2.40 wt%), whereas the acid-etched control had the lowest (16.28±3.98 wt%). Acid etching provided a reference for an intentionally altered enamel surface. However, the reported calcium comparison between the acid-etched control and the carbamide peroxide group was not statistically significant (p=0.380). Therefore, the data do not support a general statement that every bleaching protocol produced significantly less calcium alteration than acid etching, nor does this comparison establish that bleaching-related effects are clinically mild.

While all bleaching agents induced surface and mineral modifications, these alterations were uniformly less severe than those observed in the acid-etched negative control group, confirming that under controlled in vitro parameters, bleaching-induced degradation remains relatively mild. 

Limitations

This study was conducted in vitro using sectioned human teeth and therefore did not reproduce the full intraoral environment. Artificial saliva may have influenced the post-treatment elemental measurements, but the extent of this effect was not assessed. The bleaching protocols differed in several characteristics simultaneously, preventing the isolation of the effects of active ingredient, concentration, exposure duration, formulation, and light activation. While essential to simulate oral conditions, the calcium and phosphate ions present in artificial saliva promote remineralization, which partially reverses early mineral depletion and may mask subtler variations between the experimental groups [25].

## Conclusions

Within the limitations of this in vitro study, all evaluated bleaching agents induced alterations in enamel surface morphology and mineral content. The 22% carbamide peroxide gel (Pola Night) caused the most distinct demineralization and topographic changes, driven by its extended application timeline and cumulative exposure. In contrast, 37.5% hydrogen peroxide (Pola Office+) and PAP exhibited comparable, milder structural profiles. This suggests that the ultimate effect of a whitening agent on the enamel substrate is determined by a complex interplay between its chemical mechanism, formulation pH, and total clinical contact time. Phosphorus content did not differ significantly among groups. No statistically significant calcium difference was detected between the hydrogen peroxide and PAP groups, although this does not establish equivalence. These findings apply to the complete protocols tested and do not isolate the effects of exposure duration or establish clinical safety or superiority.

## References

[1] Butera A, Maiorani C, Rederiene G, Checchi S, Nardi GM (2024). Evaluation of the effectiveness of different types of professional tooth whitening: a systematic review. Bioengineering (Basel).

[2] Dewiyani S, Binarti I, Ergitamanda K (2023). Potential outcome, enamel surface roughness and tooth sensitivity of in office bleaching and at home bleaching. Int J Pharm Bio-Med Sci.

[3] Teles Junior M, Rodrigues CA, Bernardes VL, de Araujo TS, Nicoli GA, Derceli JD (2018). Dental bleaching and new possibilities: literature review. Health Sci J.

[4] Hattab FN (2024). Tooth discoloration: causes and clinical presentation-part I. J Oral Health Comm Dent.

[5] Nanjundasetty JK, Ashrafulla M (2016). Efficacy of desensitizing agents on postoperative sensitivity following an in-office vital tooth bleaching: a randomized controlled clinical trial. J Conserv Dent.

[6] Ahmed NF (2019). In vitro assessment of human enamel surface composition bleached with two different bleaching agents. Saudi J Oral Sci.

[7] Karimi Z, Saoui H, Sakout M, Abdallaoui F (2021). Effect of vital bleaching on micromorphology of enamel surface: an in vitro study. Prim Dent J.

[8] Aragão WA, Chemelo VS, Alencar CM (2024). Biological action of bleaching agents on tooth structure: a review. Histol Histopathol.

[9] Gunjal DJ, Patel PA, Bohora AA, Sharma VJ, Sharma SM (2026). Effect of carbamide peroxide and bromelain bleaching agents by the activation of diode laser on the tooth at different time intervals: an in vitro study. J Conserv Dent Endod.

[10] Coceska E, Gjorgievska E, Coleman NJ, Gabric D, Slipper IJ, Stevanovic M, Nicholson JW (2016). Enamel alteration following tooth bleaching and remineralization. J Microsc.

[11] Yusof EM, Abdullah SA, Mohamed NH (2020). Influence of light and laser activation of tooth bleaching systems on enamel microhardness and surface roughness. J Conserv Dent.

[12] Pascolutti M, de Oliveira D (2021). A radical-free approach to teeth whitening. Dent J (Basel).

[13] Yu D, Gao S, Min J, Zhang Q, Gao S, Yu H (2015). Nanotribological and nanomechanical properties changes of tooth after bleaching and remineralization in wet environment. Nanoscale Res Lett.

[14] Suresh S, Navit S, Khan SA, Sharma A, Jabeen S, Grover N, Alia S (2020). Effect of diode laser office bleaching on mineral content and surface topography of enamel surface: an SEM study. Int J Clin Pediatr Dent.

[15] de Oliveira Duque CC, Soares DG, Basso FG, Hebling J, de Souza Costa CA (2017). Influence of enamel/dentin thickness on the toxic and esthetic effects of experimental in-office bleaching protocols. Clin Oral Investig.

[16] Mena-Serrano A, Sanchez S, Granda-Albuja MG (2024). Hydrogen peroxide in the pulp chamber and color change in maxillary anterior teeth after in-office bleaching. Braz Dent J.

[17] Singh N, Ranjan D, Mahreen S, Pattanaik A, Kaur G, Nagpal A (2023). A comparative evaluation of the effects of three remineralizing agents on bleached enamel: a scanning electron microscopy (SEM) analysis. Cureus.

[18] Gokulnath R, Kumar RS, Jayasenthil A, Anjana R, Vidya GS (2025). Evaluation of bleaching efficiency of carbamide peroxide applied on different dental surfaces: an in vitro study. J Conserv Dent Endod.

[19] Saluja I, Shetty N, Shenoy R, Pangal SN (2022). Evaluation of the efficacy of diode laser in bleaching of the tooth at different time intervals using spectrophotometer: an in vitro study. J Conserv Dent.

[20] Kaur A, Waghmare P, Dodwad V (2024). Evaluation and comparison of the efficacy of teeth whitening system containing phthalimido peroxy caproic acid in conjunction with and without 810nm diode laser: a split-mouth study. Indian J Nat Sci.

[21] Abe AT, Youssef MN, Turbino ML (2016). Effect of bleaching agents on the nanohardness of tooth enamel, composite resin, and the tooth-restoration interface. Oper Dent.

[22] Llena C, Saenz L, Ghilotti J, Folguera S, Melo M (2026). Phthalimidoperoxycaproic acid (PAP) versus peroxides and impact on dental enamel after whitening treatment: an in vitro study. J Funct Biomater.

[23] Müller-Heupt LK, Wiesmann-Imilowski N, Kaya S (2023). Effectiveness and safety of over-the-counter tooth-whitening agents compared to hydrogen peroxide in vitro. Int J Mol Sci.

[24] Bhutani N, Venigalla BS, Patil JP, Singh TV, Jyotsna SV, Jain A (2016). Evaluation of bleaching efficacy of 37.5% hydrogen peroxide on human teeth using different modes of activations: an in vitro study. J Conserv Dent.

[25] Zeczkowski M, Tenuta LM, Ambrosano GM, Aguiar FH, Lima DA (2015). Effect of different storage conditions on the physical properties of bleached enamel: an in vitro vs. in situ study. J Dent.

